# External Otitis: An Unusual Presentation in Neonates

**DOI:** 10.1155/2016/7381564

**Published:** 2016-09-14

**Authors:** Peymaneh Alizadeh Taheri, Shima Rostami, Manelie Sadeghi

**Affiliations:** Pediatrics Department, Tehran University of Medical Sciences, Tehran, Iran

## Abstract

Acute otitis externa (AOE) is an infection of the external auditory canal, the auricle, and the outer surface of the tympanic membrane. Although AOE is one of the most common otologic conditions encountered in pediatric population, it is known to primarily affect children older than 2 years. We report a case of AOE caused by* Staphylococcus aureus* in a 23-day-old neonate. A 23-day-old female infant presented to our neonatology clinic with irritability and discharge from the right ear. There were yellow otorrhea, mild erythema, and edema of right external ear canal. There was no sign of otitis media on otoscopy. The results of laboratory tests were insignificant. The discharge culture grew colonies of methicillin-sensitive* Staphylococcus aureus*. After 48 hours of treatment with intravenous cloxacillin, significant improvement was observed. The present case highlights an unusual presentation of staphylococcal infection in a neonate. This is the first case of methicillin-sensitive* Staphylococcus aureus* otitis externa in an immunocompetent newborn.

## 1. Introduction

Acute otitis externa (AOE) can approximately affect 1% of general population per year [1]. It is usually manifested with mild edema and erythema of the ear canal but may also present as necrotizing otitis externa, a rare condition characterized by rapid progression and destruction of the surrounding structures [2]. AOE is typically a bacterial illness mostly caused by* Pseudomonas aeruginosa* and* Staphylococcus* spp. However,* Staphylococcus* spp. seem to be more prevalent in adults [3]. Although AOE occurs in all pediatric age groups, it is mainly considered as a disease of children aged more than 2 years [2, 4, 5]. In this article we report a rare case of staphylococcal external otitis in a 23-day-old newborn.

## 2. Case Report

A 23-day-old girl presented to our neonatal clinic because of her right ear discharge. The infant was born near term (37 weeks) and was the first single of a twin pregnancy. She was born to a 30-year-old mother, gravida 1, para 1, with no history of diseases or using medicine.

**Figure 1:**
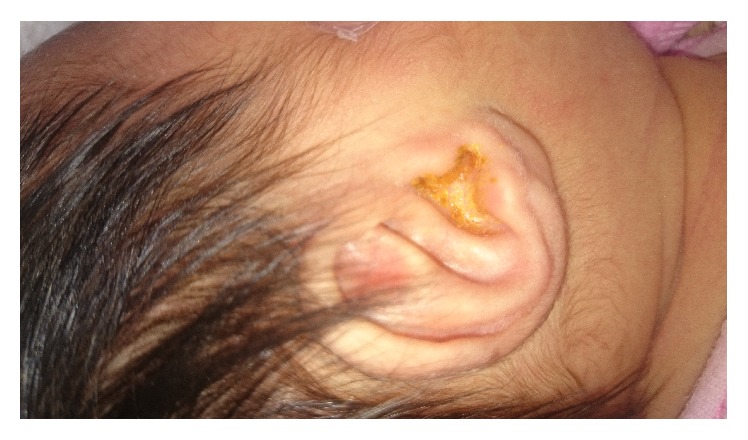
A 23-day-old girl presenting with right ear discharge.

The patient's chief complaint started with secretion from her right ear since being 19 days old. On physical examination, she had normal vital signs with axillary temperature of 36.8°C. Admission weight was 2650 gr. Her primitive reflexes were normal. There were erythema and mild edema of the right external ear canal and yellowish discharge was detected (Figure 1). There was no swelling, erythema, and cellulitis in tragus, pinna, and other surrounding tissues. All lymph nodes were intact. After administration of boric alcohol and cleaning the canal, there was no sign of otitis media on otoscopy.

Hematological investigations revealed total leucocyte count (TLC) 10300/mm^3^ with 76% neutrophil and 58% lymphocyte and no band cell, hemoglobin (Hb) 13.9 gr/dL, platelet count 374x 1000/mm^3^, C-reactive protein (CRP) 3 mg/L, and erythrocyte sedimentation rate (ESR) 2 mm/hr. Biochemical indices were normal.

The chest X-ray was normal. Blood and urine culture were negative. The discharge culture grew colonies of* S. aureus* sensitive to chloramphenicol, clindamycin, cotrimoxazole, erythromycin, vancomycin, and cloxacillin.

After 48 hours of treatment, significant improvement was observed and the neonate was discharged after 5 days of intravenous antibiotic therapy with oral cephalexin for the next 5 days. There was no recurrence on follow-up visits after 7 and 15 days.

## 3. Discussion

AOE is an infection of the external auditory canal, the auricle, and the outer surface of the tympanic membrane. The most common symptoms are otalgia, pruritis, and aural fullness [6]. On physical examination, we can find varying degrees of auditory canal erythema and edema, otorrhea, tenderness of tragus when touching it or pulling the auricle, external canal narrowing, obstruction, stenosis, or malformation [2–7]. Rarely, cranial nerve palsy, sensorineural hearing loss, or vertigo may present if the infection extends beyond the external canal [4].

AOE rarely occurs in infants and neonates [2, 4, 5]. The latest guideline published by the American Academy of Otolaryngology applies only to children aged 2 years and above. We had not encountered a case of neonatal otitis externa in our neonatology clinic or neonatal ward before. According to our search in different databases, there was no report of otitis externa during neonatal period.

The reported newborn presented with right ear otorrhea. The primary diagnosis of AOE was made based on infant's history and clinical manifestations. On right ear otoscopy, there was otorrhea along with erythema and mild edema of the external ear canal. The tragus was painful when touched and there was no structural malformation of external ear canal. The tympanic membrane was intact. There was no eczema or seborrheic dermatitis of the canal. The infant had no history of preterm birth, underlying conditions, or immunodeficiency. Our diagnosis was further confirmed by the result of microbiological culture, which revealed methicillin-sensitive* S. aureus* colonies.

It seems that* S. aureus* colonization and warm, dark, and moist canal were the probable risk factors in this case. The condition responded well to intravenous cloxacillin and local antiseptic washing (boric alcohol).
